# Molecular Characterization of Three PRORP Proteins in the Moss *Physcomitrella patens*: Nuclear PRORP Protein Is Not Essential for Moss Viability

**DOI:** 10.1371/journal.pone.0108962

**Published:** 2014-10-01

**Authors:** Chieko Sugita, Yoshihiro Komura, Korechika Tanaka, Kazuki Kometani, Hiroyuki Satoh, Mamoru Sugita

**Affiliations:** 1 Center for Gene Research, Nagoya University, Nagoya, Japan; 2 Department of Biomolecular Science, Toho University, Funabashi, Japan; Max-Planck-Institute for Terrestrial Microbiology, Germany

## Abstract

RNase P is a ubiquitous endonuclease that removes the 5′ leader sequence from pre-tRNAs in all organisms. In *Arabidopsis thaliana*, RNA-free proteinaceous RNase Ps (PRORPs) seem to be enzyme(s) for pre-tRNA 5′-end processing in organelles and the nucleus and are thought to have replaced the ribonucleoprotein RNase P variant. However, the evolution and function of plant PRORPs are not fully understood. Here, we identified and characterized three PRORP-like proteins, PpPPR_63, 67, and 104, in the basal land plant, the moss *Physcomitrella patens*. PpPPR_63 localizes to the nucleus, while PpPPR_67 and PpPPR_104 are found in both the mitochondria and chloroplasts. The three proteins displayed pre-tRNA 5′-end processing activity *in vitro*. Mutants with knockout (KO) of the *PpPPR_63* gene displayed growth retardation of protonemal colonies, indicating that, unlike Arabidopsis nuclear RPORPs, the moss nuclear PpPPR_63 is not essential for viability. In the KO mutant, nuclear-encoded tRNA^Asp^ (GUC) levels were slightly decreased, whereas most nuclear-encoded tRNA levels were not altered. This indicated that most of the cytosolic mature tRNAs were produced normally without proteinaceous RNase P-like PpPPR_63. Single *PpPPR_67* or *104* gene KO mutants displayed different phenotypes of protonemal growth and chloroplast tRNA^Arg^ (ACG) accumulation. However, the levels of all other tRNAs were not altered in the KO mutants. In addition, *in vitro* RNase P assays showed that PpPPR_67 and PpPPR_104 efficiently cleaved chloroplast pre-tRNA^Arg^ (CCG) and pre-tRNA^Arg^ (UCU) but they cleaved pre-tRNA^Arg^ (ACG) with different efficiency. This suggests that the two proteins have overlapping function but their substrate specificity is not identical.

## Introduction

RNase P is an endonuclease that removes the 5′ leader sequence from precursor tRNAs (pre-tRNAs). This endonucleolytic cleavage is an essential step in the production of mature tRNAs in all organisms, as well as in mitochondria and chloroplasts [1], [2]. Bacterial, archaeal, and eukaryotic nuclear RNase P enzymes are ribonucleoprotein complexes composed of a catalytic RNA component and one or several proteins [3]. Therefore, it was believed that 5′-maturation of tRNAs in all organisms is catalyzed by ubiquitous ribonucleoprotein (RNP) RNase P enzymes. However, human mitochondrial RNase P was identified as an RNA-free enzyme composed of three proteins called mitochondrial RNase P protein 1 (MRPP1), MRPP2, and MRPP3 [4]. MRPP3 contains two RNA-binding pentatricopeptide repeat (PPR) motifs and a conserved NYN metallonuclease domain [5], which are involved in the catalytic activity of mitochondrial RNase P. In a model plant, *Arabidopsis thaliana*, three MRPP3 homologs have been identified as RNase P enzymes; they are termed proteinaceous RNase P 1 (PRORP1), PRORP2, and PRORP3 [6]. *In vitro* cleavage assays using recombinant PRORP proteins have demonstrated that three PRORP proteins display RNase P activity [6], [7]. PRORPs are single-protein RNase P enzymes, whereas human mitochondrial RNase P is a multisubunit enzyme. PRORP1 localizes in both chloroplasts and mitochondria, whereas PRORP2 and 3 localize in the nucleus. *PRORP1* is an essential gene, because *prorp1* mutants are embryonic lethal [6]. Single *PRORP*2 or *PRORP3* knockout mutant lines show wild-type phenotypes, whereas the homozygous double mutation in *prorp2 prorp3* results in embryonic lethality [7]. This indicates that PRORP2 and 3 have redundant functions and are essential for embryogenesis and plant growth.

The Arabidopsis and rice genomes do not encode RNase P RNA, whereas they do code for several homologs of RNP RNase P protein subunits found in mammals and yeasts [8]. The presence of ribonucleoprotein RNase P enzyme in plants remains obscure. Recently, Gutmann et al. (2012) suggested that Arabidopsis had entirely replaced the ribonucleoprotein RNase P enzyme by PRORP enzymes for tRNA maturation during plant evolution [7]. However, functional characterization of PRORP proteins is limited in Arabidopsis.

Unlike in land plant organelles, an RNA subunit of bacterial-type RNase P is encoded in the organellar genomes of the Glaucophyta (*Cyanophora paradoxa*) [9], the red alga *Poryphyra purpurea* [10], and green algae (*Nephroselmis olivacea*, *Ostreococcus tauri* [11], [12]. These algae also possess a single nuclear gene for PRORP [6]. In *C. paradoxa*, RNase P activity in an extract of the photosynthesis organelle (cyanelle) is sensitive to micrococcal nuclease [13], [14], indicating the existence of bacterial-type RNase P. The nuclear-encoded PRORP in the green alga *O. tauri* has been shown to cleave the 5′ leader of pre-tRNA *in vitro* [12], [15]. Thus, algae seem to possess both a ribonuceoprotein RNase P and a PRORP. However, the latter is not known to localize in either the organelles or the nucleus.

From these differences in the status of PRORP enzymes in algae and plants, the following questions have arisen: When were plant PRORP genes duplicated during plant evolution, which PRORP protein was targeted to the organelles and which to the nucleus, and was ribonucleoprotein RNase P enzyme replaced by PRORP in plants? To answer these questions, we focused on PRORPs of the basal land plant bryophyte *Physcomitrella patens*. Here, we report the molecular characterization of three *P. patens* PRORP-like proteins (PpPPR_63, 67, and 104) that are members of the PPR protein family [16], [17]. We also report the disruption mutants of each gene and show that nuclear-localized PpPPR_63 is dispensable for moss viability and that organelle-localized PpPPR_67 and PpPPR_104 have functions that are redundant, but not completely so.

## Results

### The moss *P. patens* has three PRORP homologs

**Figure 1:**
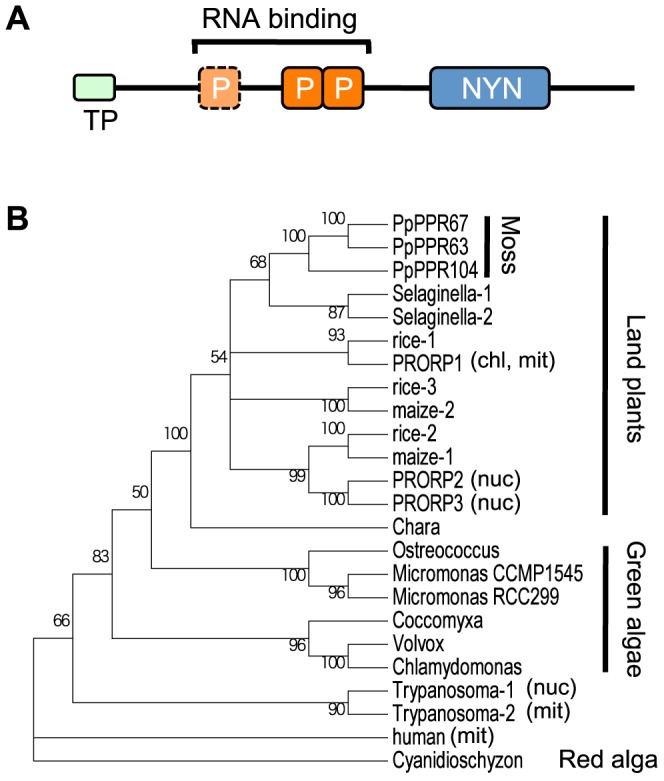
PRORP proteins are widely distributed among eukaryotes. (**A**) Schematic diagram of plant organellar PRORPs with a transit peptide (TP), RNA binding PPR motifs (P), and NYN metallonuclease domain (NYN). (**B**) Neighbor-joining phylogenetic tree of PRORPs and PRORP-like proteins. Representative PRORP protein sequences from evolutionarily distant plants were used for the phylogenic analysis. Experimentally determined localization of PRORPs is presented as chloroplast (chl), mitochondria (mit), or nucleus (nuc). Bootstrap values>50 are indicated along branches. Protein ID or accession numbers of PRORPs and PRORP-like proteins are listed in Table S1.

Among 105 *Physcomitrella* PPR proteins [17], PpPPR_63 (protein ID, Phypa_174001), PpPPR_67 (Phypa_177191) and PpPPR_104 (Phypa_152956) appeared to be PRORP homologs (Fig. 1A), because these three PPR proteins contained at least two PPR motifs and an NYN metallonuclease domain and had 42%–53% amino acid identity with Arabidopsis PRORP proteins. Although algae and *Chara* have a single PRORP homolog, mosses and vascular plants have two or three PRORP homologs (Fig. 1B and Table S1). This suggests that PRORP genes were duplicated after the emergence of land plants.

### PpPPR_63 is localized in the nucleus, whereas PpPPR_67 and 104 are found in the organelles

**Figure 2:**
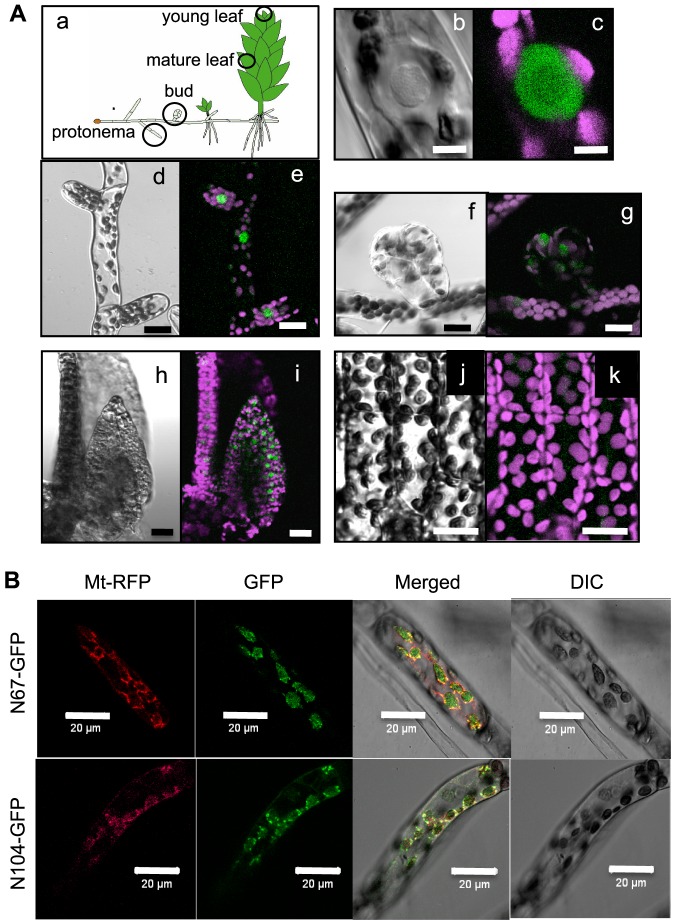
Localization of PpPPR_63, 67, and 104. (**A**) Observations of GFP fluorescence in various cells and tissues of the stable transgenic moss KI-10 line expressing PpPPR_63-GFP fusion protein. Schematic diagram of a moss plant and observed parts are in circles (a). Images are differential interference contrast (DIC) (b, d, f, h, j) and GFP (green) and chlorophyll (magenta) fluorescence (c, e, g, i, k). Single protonemal cell (b, c), protonemal cells (d, e), a bud (f, g), a young leaf (h, i), and mature leaf cells (j, k). Scale bars  = 5 µm (b, c) or 20 µm (d–k). (**B**) Subcellular localization of N-terminal PpPPR_67-GFP fusion protein (N67-GFP, upper panels) or N-terminal PpPPR_104-GFP fusion protein (N104-GFP, bottom panels). For mitochondrial localization control, the γ subunit of mitochondrial ATPase and RFP fusion protein (Mt-RFP) was used. Fluorescence of GFP and RFP, the overlay of the two fluorescence images (Merged), and the corresponding Nomarski images (DIC) are shown.

PpPPR_67 and 104 possess an N-terminal transit peptide-like sequence targeting the organelles, whereas PpPPR_63 does not. This suggests that PpPPR_67 and 104 are likely organelle-localized PRORP1 homologs, whereas PpPPR_63 seems to be a nuclear-localized PRORP2/PRORP3 homolog. To confirm this prediction we generated PpPPR_63-green fluorescent protein (GFP) knockin (KI) mosses. The four independent KI lines obtained showed normal growth (Fig. S1), and GFP fluorescence was observed in the nuclei (Fig. 2A, b and c). GFP fluorescence was observed in the protonemal cells, buds, and young leaves (Fig. 2A, d–i) but not in mature leaves (Fig. 2A, j and k).

To verify the organellar localization of PpPPR_67 and 104, their N-terminal sequences fused to GFP (N67-GFP and N104-GFP) were transiently expressed in the protonemata (Fig. 2B). GFP fluorescence of N67-GFP and N104-GFP was observed in both mitochondria and chloroplasts, coinciding with mitochondria-localized red fluorescent protein (Mt-RFP) signals and Nomarski images of chloroplasts (DIC).

### PpPPR_63, 67, and 104 have RNase P activity

**Figure 3:**
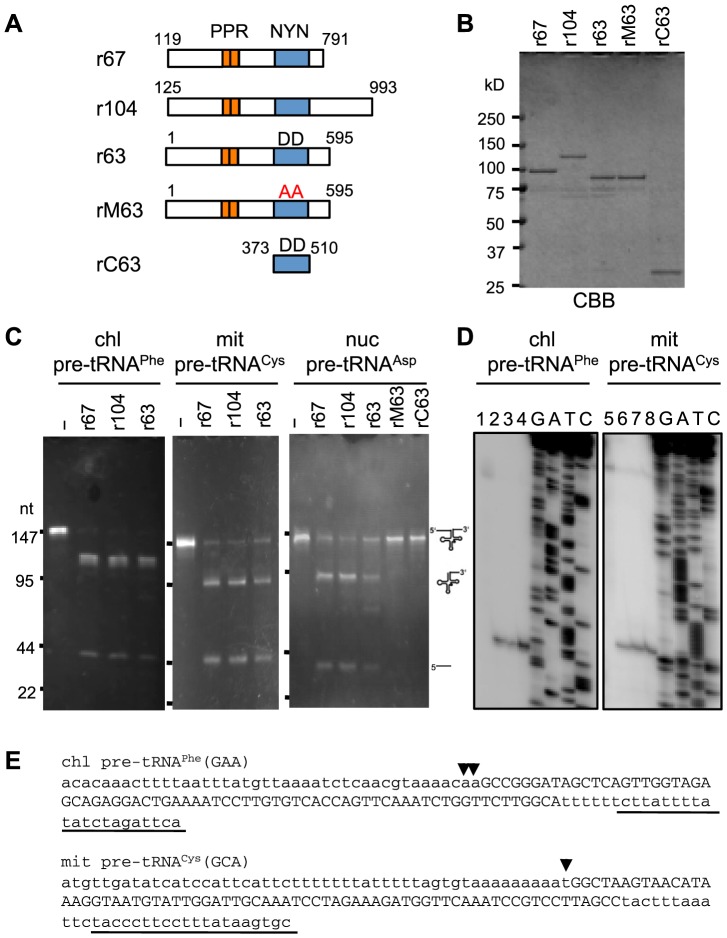
Pre-tRNA cleavage assay of *P. patens* PRORP-like proteins. (**A**) Schematic diagrams of various recombinant proteins. Recombinant proteins r67, r104, r63, and rM63 indicate, respectively, PpPPR_67, PpPPR_104, PpPPR_63, and the mutant PpPPR_63, the two catalytic aspartates (DD) of which were substituted with alanines (AA). rC63 consists of the tag sequences and an NYN domain only of PpPPR_63. (**B**) Coomassie brilliant blue (CBB)-stained recombinant proteins (1 µg each). (**C**) RNase P activity was assayed by using 2 µg of *in vitro*-transcribed chloroplast (chl) pre-tRNA^Phe^, mitochondrial (mit) pre-tRNA^Cys^, or nuclear (nuc) pre-tRNA^Asp^ and 100 ng of recombinant proteins. The reaction products were separated by using 8% denaturing PAGE and stained with ethidium bromide. (**D**) The 5′-ends of processed pre-tRNAs in (C) were determined by primer extension analysis. Processed pre-tRNAs without proteins (lanes 1 and 5), with r67 (lanes 2 and 6), with r104 (lanes 3 and 7), or with r63 (lanes 4 and 8) were reverse transcribed from the 5′-end-labeled primers underlined in (E). (**E**) The nucleotide sequences of chloroplast (chl) pre-tRNA^Phe^ and mitochondrial (mit) pre-tRNA^Cys^ are shown with small letters (5′ leader and 3′ trailer sequences) and capital letters (predicted mature tRNA). Arrowheads indicate the 5′-end positions determined in (D). Underlined sequences indicate the position of primers used for primer extension.

To investigate whether the three *Physcomitrella* PRORP-like PPR proteins are RNase P enzymes, we performed *in vitro* RNase P assays with recombinant proteins composed of the respective PRORP-like protein fused with thioredoxin at the N-terminus and six histidines and a VP5 tag at the C-terminus (Fig. 3A, B). The three recombinant proteins, r67, r104, and r63, removed the 5′ leader sequences from chloroplast pre-tRNA^Phe^, mitochondrial pre-tRNA^Cys^, and nuclear pre-tRNA^Asp^ (Fig. 3C). The 5′-end of the tRNA produced was verified by primer extension analysis (Fig. 3D, E). We also confirmed the precise cleavage of pre-tRNAs by circular reverse transcription (cRT)-polymerase chain reaction (PCR) analysis (data not shown). To verify the RNase P activity of these proteins, we also tested two recombinant proteins, rM63 and rC63. rM63 possessed a mutated NYN domain, the two adjacent aspartates of which (D_460_ and D_461_) were altered to alanines. These two aspartates have been shown to be essential for the activity of *Arabidopsis* PRORP1 [6]. The rC63 was composed of the tag sequences and the NYN domain only. The rM63 and rC63 proteins did not cleave nuclear-encoded pre-tRNA^Asp^ (Fig. 3C). These results indicated that the three moss PPR proteins had RNase P activity similar to that of Arabidopsis PRORPs [6], [7], [18].

### Nuclear PpPPR_63 is dispensable for viability

**Figure 4:**
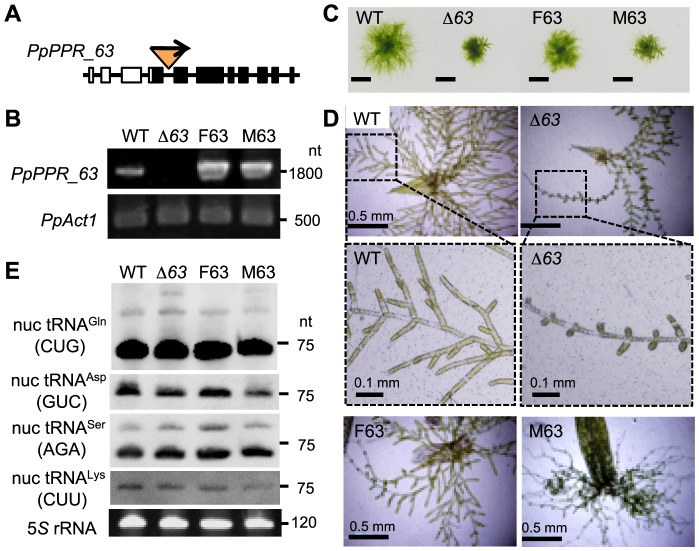
Molecular and morphological phenotypes of the *PpPPR_63* KO and complementation mosses. (**A**) Schematic diagram of *PpPPR_63* and construction of the KO locus. The *nptII* cassette was inserted into the 4th intron of the *PpPPR_63* gene. (**B**) Detection of *PpPPR_63* transcripts by RT-PCR. (**C**) Two-week-old protonemal colonies of the *PpPPR_63* KO line (Δ*63*) and the complementation lines (F63 and M63). Bars  = 5 mm. (**D**) One-week-old filamentous protonemata regenerated from cut leaves. (**E**) Steady-state levels of nuclear-encoded tRNAs.

To investigate the *in vivo* function of PpPPR_63, we generated two independent knockout (KO) mutants of *PpPPR_63*, Δ*63*-1 andΔ*63*-15. Both KO lines were verified to be null mutants. Gene structure of theΔ*63*-1 line is given as Figs. 4A and S2, and its absence of *PpPPR_63* transcripts was verified by reverse transcription (RT)-PCR (Fig. 4B). Therefore, we used theΔ*63*-1 line for further analyses (hereafter named Δ*63*). In addition, to generate the complementation lines of theΔ*63*-1, we transformed the Δ*63* moss with a transgene encoding the full length of PpPPR_63 or the mutated version and generated F63 and M63, respectively (Fig. S3). In M63, two aspartates (D_460_ and D_461_) in the NYN domain were mutated to alanines (A_460_ and A_461_). In the complementation lines F63 and M63, the introduced transgene was overexpressed (Fig. 4B). The KO mutantΔ*63* and the complementation line M63 mosses displayed smaller protonemal colonies than the wild type (WT) (Fig. 4C), and they showed abnormal regeneration from leaves (especially defective growth of the sub-apical cells) (Fig. 4D). The complementation line F63 exhibited a phenotype the same as that of the WT. These results indicated that *PpPPR_63* was responsible for the mutant phenotype.

### Knockout of *PpPPR_63* slightly affects specific nuclear-encoded tRNAs

If nuclear PpPPR_63 could function as a *bona fide* RNase P, knockout of the *PpPPR_63* gene could result in severely reduced accumulation of nuclear-encoded tRNAs. To investigate this possibility, we analyzed the accumulation levels of several nuclear-encoded tRNAs in theΔ*63* mutant. For this analysis nuclear-encoded tRNA^Asp^ (GUC) and tRNA^Gln^ (CUG) were chosen, because expression of these tRNAs has been demonstrated to significantly decrease in the knockdown mutants of nuclear PRORP2/3 plants [7]. tRNA^Asp^ (GUC) in theΔ*63* mutant and M63 complementation line was slightly reduced to 70% of that in the WT and F63. Whereas, tRNA^Gln^ (CUG) in the Δ*63* mutant, M63 and F63 complementation lines accumulated to the same level as in the WT (Fig. 4E). Similarly, nuclear-encoded tRNA^Ser^ (AGA) and tRNA^Lys^ (CUU) levels were not affected in the mutants. This suggests that PpPPR_63 PNase P activity may be limited to specific tRNA(s) including tRNA^Asp^ (GUC).

### Knockout of *PpPPR_104* results in a significant reduction in chloroplast tRNA^Arg^ (ACG)

**Figure 5:**
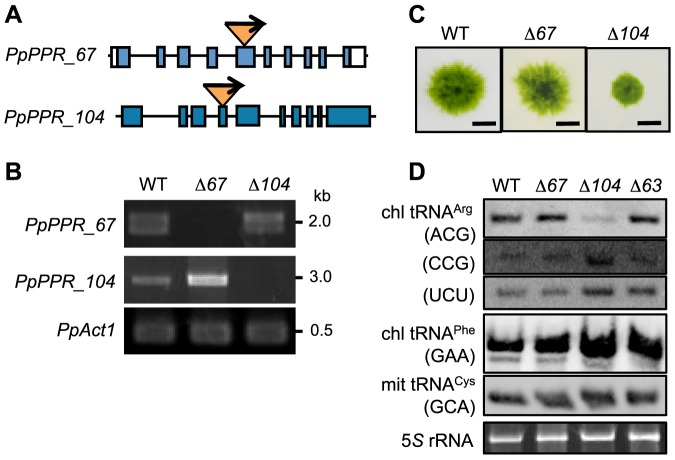
*PpPPR_67* and *PpPPR_104* KO mosses. (**A**) Schematic structure of *PpPPR_67* and *104* KO loci. The *nptII* cassette was inserted into the 5th exon of *PpPPR_67* and the 4th exon of *PpPPR_104*. (**B**) Detection of *PpPPR_67* and *104* transcripts by RT-PCR. (**C**) Two-week-old protonemata colonies of the *PpPPR_67* KO line (Δ*67*) and the *PpPPR_104* KO line (Δ*104*). Scale bars  = 5 mm. (**D**) Steady-state levels of chloroplast (chl) tRNA^Arg^ isoacceptors with different anticodons, chl tRNA^Phe^ (GAA) and mitochondrial (mit) tRNA^Cys^ (GCA).

To investigate the *in vivo* function of the *PpPPR_67* and *104* genes, we generated and characterized their KO mutants (Figs. 5A, S4, S5, S6). The single gene KO mutants obtained, Δ*67* andΔ*104*, were null mutants (Fig. 5B), and the protonemal colony ofΔ*104* moss was smaller than that of the WT (Fig. 5C). A peunumbra and central part of the protonemal colony mostly consists of caulonemal and chloronemal filaments, respectively. In theΔ*104* moss, caulonemal filaments were poorly induced, resulting in a smaller colony. In contrast, *PpPPR_67* KO moss (Δ*67*) showed little difference from the WT. To generate double KO mutants, we carried out four times transformation experiments, such as introducing the PpPPR_104 KO plasmid into the *PpPPR_67* KO line (Δ*67*-1-17 line) and vice versa. However, no double KO mutants were obtained from the genotyping of 262 moss plants. This indicates that *PpPPR_67* and *104* genes cannot be knocked out simultaneously. Since they may have redundant functions, single KO mutants are viable but double KO is probably lethal.

To investigate whether organellar tRNAs were affected in the *PpPPR_67* and *104* KO mutants, we performed northern blot analyses by using all organelle-encoded tRNA probes. The chloroplast tRNA^Arg^ (ACG) level significantly decreased inΔ*104* but not inΔ*67* orΔ*63* (Fig. 5D). All the other tRNAs, including chloroplast tRNA^Phe^ (GAA) and mitochondrial tRNA^Cys^ (GCA) that were used for the *in vitro* RNase P cleavage assay, were not altered in the WT and the three KO mutants (Figs. 5D, S7, S8). This result suggests that PpPPR_67 and PpPPR_104 have redundant functions as RNase P in moss organelles.

### PpPPR_67 and 104 cleave chloroplast pre-tRNA^Arg^ (ACG) with different efficiency

**Figure 6:**
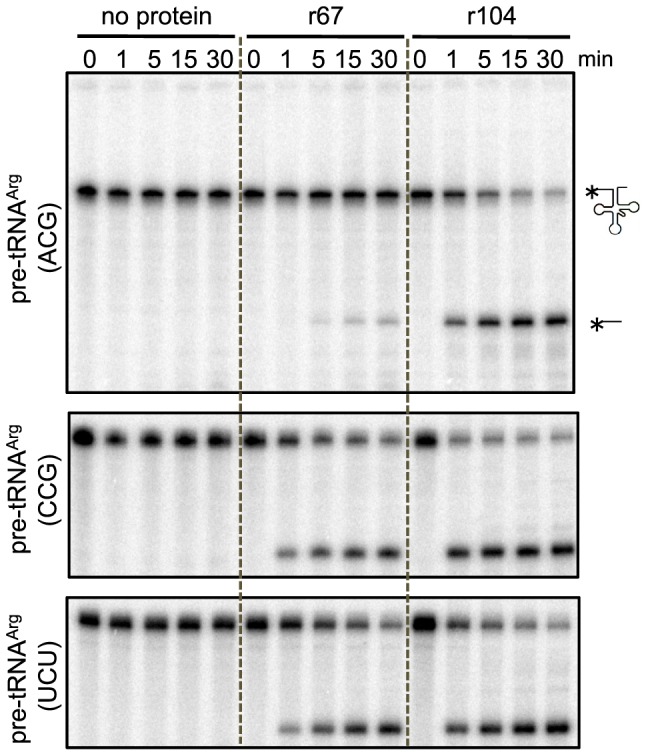
Pre-tRNA cleavage assay of the recombinant proteins r67 and r104. Reactions containing 500 nM of chloroplast 5′-^32^P-pre-tRNA^Arg^ (ACG) (upper), tRNA^Arg^ (CCG) (middle), or tRNA^Arg^ (UCU) (lower) and 500 nM of recombinant protein were quenched at 1, 5, 15, and 30 min points, resolved on 8% denaturing PAGE, and analyzed with a phosphoimager.

Because chloroplast tRNA^Arg^ (ACG) levels decreased in the Δ*104* but not in theΔ67, we assumed that PpPPR_104 and PpPPR_67 would have distinct RNase P activity against chloroplast pre-tRNA^Arg^ (ACG). To test this possibility, three chloroplast pre-tRNA^Arg^ isoacceptors were used as substrates for *in vitro* RNase P cleavage assay. This assay revealed that PpPPR_67 and 104 efficiently cleaved pre-tRNA^Arg^ (CCG) and pre-tRNA^Arg^ (UCU) but they cleaved pre-tRNA^Arg^ (ACG) with different efficiency (Fig. 6). These results suggest that the function of their RNase P is not completely redundant.

## Discussion

The first two questions to be addressed here were: When were plant *PRORP* genes duplicated during plant evolution and where were the respective PRORP proteins localized or targeted to? Our present study provides evidence that *PRORP* genes were duplicated after the emergence of the early land plants, i.e. mosses. The moss *P. patens* has three PRORP-like proteins displaying RNase P activity *in vitro*. Arabidopsis PRORP1 is localized in both the mitochondria and chloroplasts, and PRORP2 and 3 are localized in the nucleus [6]. On the other hand, the moss *P. patens* has two PRORP1-like proteins, PpPPR_67 and 104, both of which are dual-targeted to the mitochondria and chloroplasts, and one nuclear-localized PRORP2/3-like PpPPR_63. Thus, duplicated *PRORP* gene products are likely targeted to either the organelles or the nucleus.

### Nuclear PpPPR_63 is involved in normal plant growth and branch formation of protonemata

In Arabidopsis, the double KO mutation of *PRORP2* and *3* resulted in embryonic lethality [6]. In contrast, a null mutant of *PpPPR_63* displayed smaller protonemal colonies than the WT and also showed abnormal branch formation of filamentous protonemata (Fig. 4). This indicates that unlike Arabidopsis nuclear RPORPs, the moss nuclear PpPPR_63 is dispensable for plant viability. The nuclear-encoded tRNA^Asp^ (GUC) level in the *PpPPR_63* KO mutant was slightly decreased (to 70% of that in WT mosses), but most nuclear-encoded tRNA levels were not altered. This result indicates that most of the cytosolic mature tRNAs were produced normally, without proteinaceous RNase P-like PpPPR_63. This does not concur with the results observed from functional analysis of Arabidopsis PRORP2 and 3 [6], [7]. In Arabidopsis mutant plants where PRORP3 was absent and the PRORP2 level was decreased, the steady-state levels of five nuclear-encoded tRNAs investigated were significantly reduced, to 30% of the tRNA levels in control plants [7]. These observations indicate that Arabidopsis nuclear PRORP2 and 3 function as master RNase P enzymes, while the moss PpPPR_63 is not the sole RNase P enzyme in the nucleus.

It is unlikely that the slight reduction of the tRNA^Asp^ (GUC) level resulted in growth retardation and abnormal branch formation in the *PpPPR_63* KO mutant. PpPPR_63 may have a versatile function rather than pre-tRNA 5′-end processing. Arabidopsis PRORP proteins are involved in the maturation of not only tRNAs but also mRNA or snoRNA [7]. Similarly, PpPPR_63 is probably involved in the maturation of unknown mRNA or small RNA-containing tRNA-like structures.

### Organelle-localized PpPPR_67 and 104 have functions not completely redundant for tRNA accumulation

In this study, we generated single KO mutants of *PpPPR_67* and *PpPPR_104* but failed to obtain double KO mutants. Similarly, KO mutation of *Arabidopsis PRORP1* was shown to result in embryonic lethality [6]. This suggests that PpPPR_67 and 104 have redundant functions. However, it is interesting to observe that a single mutant of *PpPPR_67* and *104* displayed different phenotypes of protonemal growth and chloroplast tRNA^Arg^ (ACG) accumulation. Chloroplast tRNA^Arg^ (ACG) significantly decreased in the *PpPPR_104* KO mutant but not in *PpPPR_67* KO mutants. The *P. patens* chloroplast genome codes for three tRNA^Arg^ isoacceptors [19]. An *in vitro* RNase P cleavage assay revealed that pre-tRNA^Arg^ (ACG) was more efficiently cleaved by PpPPR_104 than by PpPPR_67, whereas pre-tRNA^Arg^ (UCU) and pre-tRNA^Arg^ (CCG) were cleaved with similar efficiency by both proteins. This is the first evidence that PRORP enzymes have substrate specificity of tRNA processing. The anticodon of *P. patens* tRNA^Arg^ (ACG) is modified that including inosine in chloroplasts, and tRNA^Arg^ (ICG) can recognize all four codons. Chloroplast tRNA^Arg^ (CCG) recognizing only the GGC codon is dispensable [20]. Processing of chloroplast pre-tRNA^Arg^ (ACG) by PpPPR_104 is essential for the accumulation of mature tRNA^Arg^ (ACG). Therefore, the *PpPPR_104* KO mutant may have displayed small protonemal colonies because of a great reduction in mature tRNA^Arg^ (ACG) levels.

Unlike Arabidopsis [6], [7], pre-tRNAs were hardly detected in the moss organelles (Figs. S7 and S8). It is conceivable that precursors might be turned over faster than mature tRNAs in mosses.

### Was RNP-type RNase P enzyme entirely replaced by PRORP protein in mosses?

**Figure 7:**
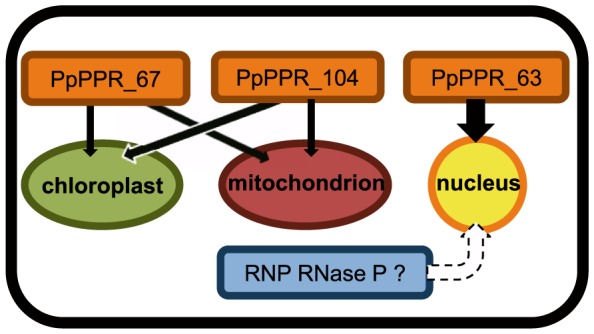
Schematic summarizing the localization of three PRORP homologs, PpPPR_63, 67, and 104 in *P. patens.* This study suggested that unidentified RNase P enzyme(s) including RNP-type RNase P may be present at least in the nucleus.

The last question is whether ribonucleoprotein RNase P enzyme was replaced by PRORP in both the organelles and the nucleus. In this study, we showed that disruption of *PpPPR_63* did not result in a significant reduction in nuclear-encoded tRNAs. This raises the possibility that unidentified RNase P activity may contribute to pre-tRNA maturation in the moss nucleus (Fig. 7). However, it remains unknown whether 5′-end processing of nuclear pre-tRNAs is supported by RNP-type RNase P. In *P. patens*, an RNA component of RNP-type RNase P was not identified, yet but protein components (Pop4, Rpp1, Pop1, and Pop5) are encoded in the genome [21]. Identification of RNase P activity rather than PRORP1-type PpPPR_63 will be a challenge for the future.

## Materials and Methods

### Accession numbers

The sequences reported in this paper have been deposited in the DDBJ/GenBank/EMBL databases with accession numbers: *PpPPR_63* mRNA, AB983707; *PpPPR_67* mRNA, AB983708; *PpPPR_104* mRNA, AB808584.

### Plant material and preparation of DNA and RNA

The moss *P*. *patens* subsp. *patens* was grown at 25°C under continuous light (30 µmol photon m^−2^s^−1^), and genomic DNA and total cellular RNA were prepared from the moss protonemata as described previously [22].

### Preparation of recombinant proteins

DNAs corresponding to the proteins (1–595 residues for PpPPR_63, 119–791 for PpPPR_67, and 125–993 for PpPPR104) were amplified by using the primers listed in Table S2 and cloned into pBAD/Thio-TOPO vector (Invitrogen). Proteins were expressed for 6 h at 18°C in *E. coli* BL21 and purified as described previously [23].

### Preparation of pre-tRNA

DNAs representing precursors of chloroplast tRNA^Phe^, mitochondrial tRNA^Cys^, and nuclear tRNA^Asp^ were amplified by PCR from *P. patens* genomic DNA with oligonucleotide pairs T7-cpF5 and cpF3, T7-mtC5 and mtC3, and T7-nuDGTC5 and nuDGTC, respectively (Table S2). The 5′ oligonucleotides contained the T7 promoter sequence. The pre-tRNA^Phe^, pre-tRNA^Cys^, and nuclear pre-tRNA^Asp^ were respectively 142, 153, and 140 nt long and were designed with respective 5′ leaders of 42, 51, and 40 nt and 3′ trailers of 27, 32, and 25 nt. Amplified DNAs (200 ng) were transcribed with T7 RNA polymerase (TaKaRa) for 1 h at 37°C, then digested with 5 U of RNase-free DNase I (TaKaRa) and 20 U of RNase Inhibitor (TaKaRa) for 15 min at 37°C. Synthesized pre-tRNAs were purified by phenol chloroform extraction.

For preparation of chloroplast pre-tRNA^Arg^ isoacceptors, the corresponding genes were cloned and *in vitro* transcribed. The obtained pre-tRNAs were dephosphorylated with Thermo Sensitive Alkaline Phosphatase (Promega) and were 5′-end radiolabeled with [γ^32^P] ATP and polynucleotidekinase (TaKaRa). Labeled RNAs were purified from the gel after 8% polyacrylamide gel electrophoresis (PAGE) containing 7M urea.

### Pre-tRNA cleavage assay

Cleavage assays were performed as described previously [7]. Recombinant protein (100 ng) and *in vitro*-synthesized pre-tRNA (2 µg) were incubated for 1 h at 25°C in a 20 µl mixture of 20 mM Tris-HCl (pH 8.0), 30 mM KCl, 4.5 mM MgCl_2_, 20 µg/ml bovine serum albumin, and 2 mM DTT. A half of the reaction mixture was separated on 8% polyacrylamide-7 M urea gels and stained with ethidium bromide.

For cleavage assays of three chloroplast pre-tRNA^Arg^ isoacceptors, the 5′ end-radiolabeled pre-tRNAs (500 nM each) and the recombinant proteins (500 nM each) were incubated and aliquots were withdrawn at the indicated times. Dried PAGE gels were analyzed by Storm 820 (GE Healthcare).

### Determination of the 5′-end of tRNA

The^ 32^P 5′-end-labeled oligonucleotides cp-F3 or mt-C3 (Table S2) and 200 ng of *in vitro*-cleaved RNAs were mixed and reverse transcribed with ReverTraAce (ToYoBo) for 1 h at 42°C, then digested with RNase A and precipitated with ethanol. The sequence ladders were obtained by using template DNA, the radiolabled primer cp-F3 or mt-C3, as described previously [22].

### Subcellular localization

The DNA region from the 6th intron to the last codon of the *PpPPR_63* gene was inserted in-frame into the GFP coding region of pGFPmutNTPII (http://moss.nibb.ac.jp). The 3′-flanking region of *PpPPR_63* was inserted downstream of the *nptII* cassette of pGFPmutNTPII. The resultant plasmid p63-GFPKI (Fig. S1) was linearized with *Not*I and introduced into *P. patens* by particle bombardment as described previously [23]. For N67-GFP and N104-GFP, cDNAs corresponding to an N-terminal part of the proteins (residues 1–112 and 1–105, respectively) were amplified by using the appropriate primers (Table S2) and cloned into pKSPGFP9 [23]. The resultant plasmid was cotransfected with pMt-RFP into *P. patens* protonemata and GFP and RFP fluorescence were observed as described previously [24].

### Generation of KO lines

The 5′ region (2960 bp) of *PpPPR_63*, a 2958-bp region of *PpPPR_67*, and a 3032-bp region of *PpPPR_104* were amplified from moss genomic DNA by PCR using the appropriate primers (Table S2) and cloned into pGEM-T Easy (Promega). The KO plasmid p63KO4-13A (Fig. S2) was generated by insertion of the *nptII* cassette into the 4th intron of the *PpPPR_63* gene by using the GPS-M mutagenesis System (NEB). The KO plasmids p67KO-1 and p67KO-2 (Figs. S4 and S5) were generated by insertion of the *nptII* cassette into *Hin*dIII in the 4th exon, or into *Stu*I in the 5th exon, respectively, of the *PpPPR_67* gene. The KO plasmid p104KO (Fig. S6) was generated by insertion of the *nptII* cassette into the *Bgl*II site in the 4th exon of the *PpPPR_104* gene. These KO plasmids were digested with *Not*I and introduced into *P. patens* by particle bombardment.

### Generation of complementation lines

For F63, the *PpPPR_63* cDNA coding region (1785 bp) amplified by PCR using specific primers (Table S2) was cloned into the *Swa*I site of the overexpression vector pOX9WZ1 [25]. The mutated *PpPPR_63* (M63: D_460_A/D_461_A) was amplified from the F63 construct by using the primers 63DA-F and 63DA-R. Both constructs were linearized with *Not*I and introduced into the KO line Δ*63*.

### Northern blot analysis

Total cellular RNA (5 µg) was separated on 10% polyacrylamide-7 M urea gels and transferred to nylon membranes. Hybridization and washing were performed at 42°C. The gene-specific oligonucleotide probes (Table S2) were labeled with DIG-ddUTP and terminal deoxynucleotidyl transferase (Roche). Signals were acquired with EZ-capture (ATTO).

## Supporting Information

Figure S1 **Generation of** ***PpPPR_63*** **-** ***GFP*** **knockin (KI) mosses.** (**A**) Schematic of insertion of p63-GFPKI into the 3′ terminal end of the *PpPPR_63* coding region. Translated and untranslated regions are represented by black and white boxes, respectively. Primers P1 to P3 used for PCR, and the expected amplified DNA sizes, are shown. (**B**) PCR analysis using the indicated primer pairs. The predicted 4.5- and 1.8-kb fragments were amplified from the KI lines. (**C**) Protonemal colonies of wild-type (WT) and KI mosses grown for 2 weeks on BCDATG medium plates without antibiotics. KI-10 was used for further analyses (Fig. 2). Scale bars  = 5 mm.
(ZIP)

Figure S2 **Generation of** ***PpPPR_63*** **knockout (KO) moss.** (**A**) Schematic diagrams of the *PpPPR_63* gene, the KO construct (p63KO4-13), and the generated knockout locus. A Geneticin G418-resistance gene (*nptII*) cassette was inserted into the 4th intron of *PpPPR_63*. Translated and untranslated regions are represented by black and white boxes, respectively. Primers P4 to P7 used for PCR and the expected amplified DNA sizes are shown. The DNA probe used in (C) is represented by grey bars. (**B**) PCR analysis using the indicated primer pairs. The predicted 1.9-, 2.2- and 5.5-kb fragments were amplified from the KO lines; 3.0 kb was amplified from the WT. (**C**) Total cellular DNAs (20 µg) from wild type (WT) and KO mosses were digested with *Sph*I and hybridized with the *PpPPR_63* probe in (A). The probe was labeled with DIG-dUTP. Predicted hybridized fragments were detected in WT and KO mosses.
(EPS)

Figure S3 **Generation of** ***PpPPR_63*** **complementation lines intoΔ** ***63*** **-1.** (**A**) Schematic diagrams of *PpPPR_63* loci of wild type andΔ*63*-1. Primers P8 and P9 and the expected fragment sizes for PCR analysis are shown. (**B**) The *PpPPR_63* coding region (1.8 kb) amplified from the wild type cDNA was cloned into the *Swa*I site of the overexpression vector, pOX9WZ1, which harbors a rice actin promoter, a c-myc tag and the Zeocin resistance cassette. The mutated *PpPPR_63* (M63: D_460_A/D_461_A) gene was modified from the F63 construct. (**C**) The predicted 1.8- and 3.0-kb fragments derived from the transgenic lines and wild type (WT), respectively, were amplified. The predicted 5.0-kb fragments were not amplified fromΔ*63*-1 under the PCR conditions. F63-3 and M63-8 plants were used for further analysis as F63 and M63, respectively, in Fig. 4. Transgenic lines V-7 and V-8 were generated by transferring the vector only into theΔ*63*-1 moss.
(EPS)

Figure S4 **Generation of** ***PpPPR_67*** **knockout (KO) lines.** (**A**) Schematic diagrams of the *PpPPR_67* gene (WT), the KO construct (p67KO-1), and the generated KO locus. A Geneticin G418-resistance gene (*nptII*) cassette was inserted into the *Hin*dIII site of the 4th exon of *PpPPR_67*. Primers used for PCR and the expected amplified DNA sizes are shown. (**B**) The predicted 5.0-, 2.1- and 1.6-kb fragments were amplified from the transgenic lines, and 3.0 kb was amplified from the WT. The KO line *67*-1-17 was used in Figs. S7 and S8.
(EPS)

Figure S5 **Generation of** ***PpPPR_67*** **knockout (KO) lines.** (**A**) Schematic diagrams of the *PpPPR_67* locus, the KO construct (p67KO-2), and the targeted KO locus. A Geneticin G418-resistance gene (*nptII*) cassette was inserted into the *Stu*I site of the 5th exon of *PpPPR_67*. Primers and the amplified DNA sizes for PCR analysis are shown. (**B**) The predicted 5.0-, 2.3- and 1.5-kb fragments were amplified from the transgenic lines, and 3.0 kb was amplified from the WT. The KO line *67*-2-2 was used asΔ*67* in Figs. 5, S7, and S8.
(EPS)

Figure S6 **Generation of** ***PpPPR_104*** **knockout (KO) line.** (**A**) Schematic diagrams of the *PpPPR_104* locus, the KO construct (p104KO), and the targeted KO locus. A Geneticin G418-resistance gene (*nptII*) cassette was inserted into the *Bgl*II site of the 4th exon of *PpPPR_104*. Primers used for PCR and the expected fragment sizes are shown. (**B**) The predicted 5.0-, 1.8- and 1.3-kb fragments were amplified from the transgenic lineΔ*104*-7, and 3.0 kb was amplified from the wild type (WT). The KO line *104*-7 was used asΔ*104* in Figs. 5, S7, and S8.
(EPS)

Figure S7 **Northern blot analysis of chloroplast tRNAs.** Total cellular RNAs (5 µg) from wild type (WT), Δ*67*-1-17, Δ*67*-2-2, andΔ*104*-7 protonemata were separated on 8% polyacrylamide containing 7 M urea and transferred to nylon membranes. Chloroplast tRNA gene-specific oligonucleotide probes (Table S2) were labeled with DIG-ddUTP and terminal deoxynucleotidyl transferase (Roche).
(TIF)

Figure S8 **Northern blot analysis of mitochondrial tRNAs.** Total cellular RNAs (5 µg) were subjected to northern blot analysis as described in Fig. S7. Mitochondrial tRNA gene-specific oligonucleotide probes (Table S2) were labeled as described in Fig. S7.
(TIF)

Table S1 **List of PRORP and PRORP-like proteins from various organisms.**
(XLSX)

Table S2 **Oligonucleotides used in this study.**
(XLSX)
