# Supplementary figures and images for: Molecular Characterization of Three PRORP Proteins in the Moss Physcomitrella patens: Nuclear PRORP Protein Is Not Essential for Moss Viability
Source: PLoS One. 2014 Oct 1;9(10):e108962. doi: 10.1371/journal.pone.0108962 (PMC4201334; doi:10.1371/journal.pone.0108962)

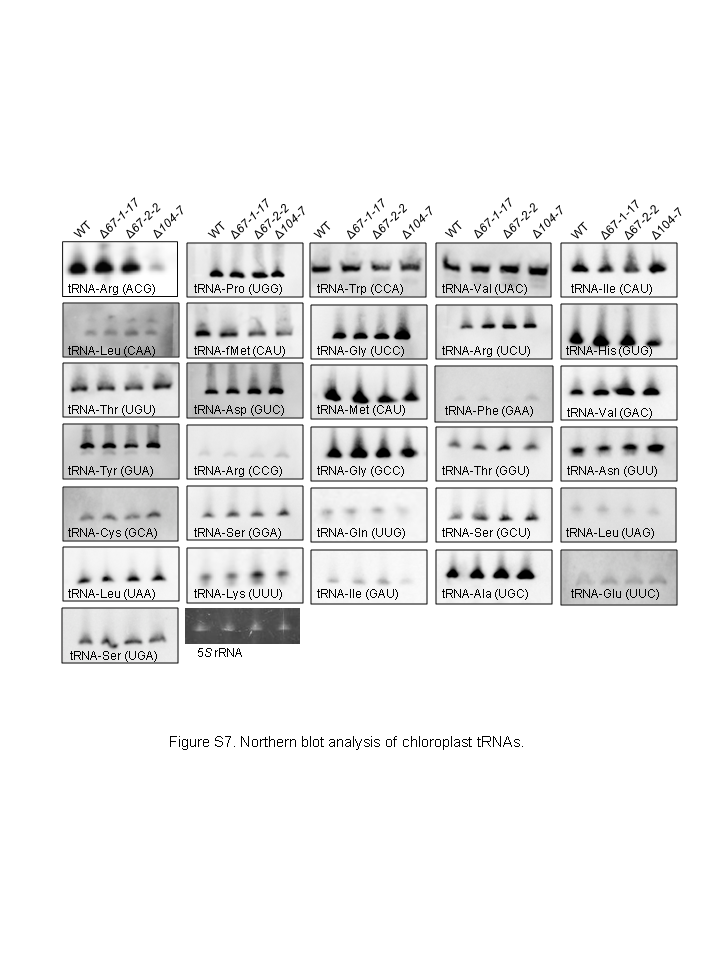

Supplement: Figure S7 — Northern blot analysis of chloroplast tRNAs. Total cellular RNAs (5 µg) from wild type (WT), Δ67-1-17, Δ67-2-2, andΔ104-7 protonemata were separated on 8% polyacrylamide containing 7 M urea and transferred to nylon membranes. Chloroplast tRNA gene-specific oligonucleotide probes (Table S2) were labeled with DIG-ddUTP and terminal deoxynucleotidyl transferase (Roche). (TIF) [file pone.0108962.s007.tif]

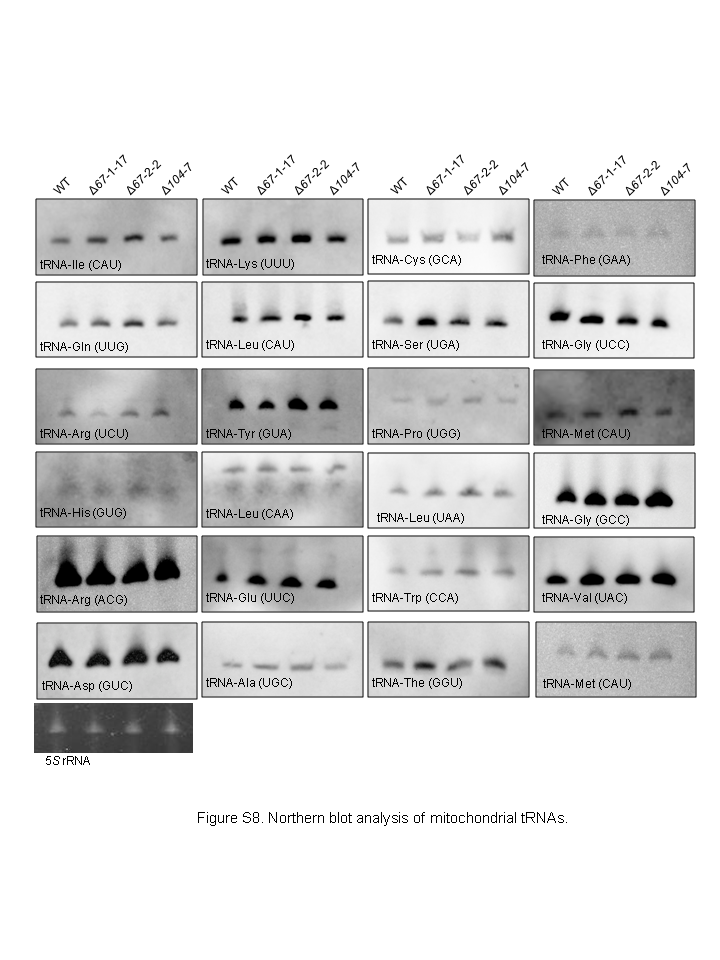

Supplement: Figure S8 — Northern blot analysis of mitochondrial tRNAs. Total cellular RNAs (5 µg) were subjected to northern blot analysis as described in Fig. S7. Mitochondrial tRNA gene-specific oligonucleotide probes (Table S2) were labeled as described in Fig. S7. (TIF) [file pone.0108962.s008.tif]
